# Is Susceptibility to Default Effects Associated with Age?

**DOI:** 10.1093/geroni/igab046.2629

**Published:** 2021-12-17

**Authors:** Julia Nolte, Corinna Loeckenhoff

**Affiliations:** Cornell University, Ithaca, New York, United States

## Abstract

Older adults are more likely to avoid making decisions than younger adults are. Because the underlying reasons are poorly understood, the present study investigated the potential role of age differences in susceptibility to default effects. Defaults facilitate decision avoidance because decision makers are more likely to passively accept than to actively reject pre-selected default options. A representative lifespan sample (N = 500, Mage = 49.90, SDage = 19.34, 51% female, 67% non-Hispanic White) responded to a pre-registered online study. Participants completed one default effect task comprising two scenarios, one requiring opt-out and one requiring opt-in decisions (i.e., 15 vs. 0 pre-selected features each). Susceptibility to defaults was assessed through the discrepancy between scenarios. In addition, we collected data on known determinants of default effect compliance (i.e., perceived endowment, endorsement, ease, importance of the choice, and experience making similar choices) as well as post-decisional affect. Finally, participants responded to assessments of demographic background, personality, socioemotional and health status, and cognitive ability. Susceptibility to default effects was evident both at the individual and the group level (i.e., across and within scenarios). Unlike hypothesized, older age did not predict greater susceptibility, and older adults were less rather than more likely to endorse determinants of default effect compliance. Of the covariates assessed, only identifying as non-Hispanic White, greater perceived endorsement, and greater perceived ease predicted decision makers’ susceptibility to default effects. Thus, results did not support our assumption that age differences in decision avoidance might reflect age-related increments in the acceptance of decision defaults.

